# Protocol and the post-human performativity of security techniques

**DOI:** 10.1177/1474474015609160

**Published:** 2015-10-09

**Authors:** Nathaniel O’Grady

**Affiliations:** University of Southampton, UK

**Keywords:** aesthetics, emergency, exercise, performativity, protocol, security

## Abstract

This article explores the deployment of exercises by the United Kingdom Fire and Rescue Service. Exercises stage, simulate and act out potential future emergencies and in so doing help the Fire and Rescue Service prepare for future emergencies. Specifically, exercises operate to assess and develop protocol; sets of guidelines which plan out the actions undertaken by the Fire and Rescue Service in responding to a fire. In the article I outline and assess the forms of knowledge and technologies, what I call the ‘aesthetic forces’, by which the exercise makes present and imagines future emergencies. By critically engaging with Karen Barad’s notion of post-human performativity, I argue that exercises provide a site where such forces can entangle with one another; creating a bricolage through which future emergencies are evoked sensually and representatively, ultimately making it possible to experience emergencies in the present. This understanding of exercises allows also for critical appraisal of protocol both as phenomena that are produced through the enmeshing of different aesthetic forces and as devices which premise the operation of the security apparatus on contingency.

## Introduction

This article focuses on the design and performance of exercises by the Fire and Rescue Service (FRS) in the United Kingdom. Exercises stage, imagine and simulate potential future fire emergencies by their sensual, spatial, temporal and audio-visual features and effects. The article also demonstrates how, by their bringing future emergencies to light in the present, exercises function to assess, develop and create forms of protocol which plan out and guide the FRS’ response to different kinds of fire emergencies, before these emergencies occur. The use of exercises to both imagine future fire emergencies and in turn to plan protocol resonates with broader organisational changes to both the FRS and the wider securing of emergencies over the last decade in the U.K. With the introduction of legislation such as the *Fire and Rescue Services Act*^1^ and the *Civil Contingencies Act*^2^ strategic emphasis in the FRS is now placed not just on responding to fires as and when they occur but also on building capacity to identify fire as a future event which can be governed in the present. Instituting these changes, the FRS follows a path noted across political geography and critical security studies which outlines how security organisations increasingly enact anticipatory forms of governance in which intervention takes place in the present but is directed towards and shaped by understandings of the possibility of emergencies in the future.^3–5^

Although heretofore unaccounted for in literature on anticipatory governance, protocol is emblematic of this turn towards risk-based, anticipatory forms of governance. Protocols are a planned sequence of actions which structure response to emergencies. As will be detailed in more depth below, different protocol exists for different types of fire emergencies. In relation to a specific type of fire emergency, protocol can, for instance, refer to instructions on how to handle equipment, guide on the distance from which fires should be extinguished or how fire fighters should avoid the dangers associated with tackling a blaze in a particular kind of space. As part of the anticipatory strategic architecture of the FRS, protocol enacts a form of preparedness by which the FRS plans their response for future fire emergencies before they occur. Response on first sight may appear as the most important component of the FRS’ responsibility. It is through response that the FRS attends to fires as they unfold after all. With the organisational changes instituted over the last decade, however, response has undergone a kind of temporal reconfiguration. Instead of merely accounting for action in the present, the response of the FRS to fires is planned out in different protocols.^6^ By allowing the FRS to prepare in the present for response to future emergencies, protocol cuts across two temporalities. At once protocol intervenes on the future because it entails planning a set of actions before an event has had the chance to occur. At the same time, protocol structures the actions taken once the future to which it is oriented unfolds as an event in the present.^7^

This article shows how exercises operate to assess and develop protocol by imagining future fire emergencies to which exercise participants respond in real time. The twin move of both assessment and development is achieved by presenting in exercises future emergencies which challenge the effectiveness of protocol which has already been established. Pushing the limit of a protocol’s applicability, as will be documented later, means acting out in exercises future emergencies which have never been experienced before. To explore exercises in terms of their remaking of protocol, I examine the forms of knowledge and technologies used in both the design and performance of exercises. Deployed together, these knowledges and technologies coordinate with one another and operate as aesthetic forces which make present future emergencies. Drawing on a burgeoning and evermore vast literature deriving from cultural and political geography,^8,9^ literary studies,^10^ politics^11^ and new media studies,^12^ the article conceptualises aesthetics not simply as the study of the principles underpinning the creation of beautiful objects. Instead, aesthetics is taken to mean the set of techniques through which different scenarios and events can be rendered, imagined and experienced. But aesthetics is used also to refer to how such events are both produced through and beheld upon sensual registers. The senses penetrated to bring the future emergency to light in exercises are various. When exercises are being designed, for example, experiential and tacit knowledge of FRS personnel works in tandem with cinema technologies to imagine the event which will be exercised, while the the performance of exercises rely on invoking different emotional states in participants through the presence of material props which entangle with the discursive and audio-visual invocation of the future emergency.

Much has been written about exercises as emblematic of the new forms of knowledge which are harnessed and deployed as agents of the security apparatus think of their operation as to anticipate future events.^13,14^ In recognition of the uncertainty that inhabits the future, it has been argued that cold statistics made sense of through probability logics are not the only forms of knowledge that circulate across a security apparatus increasingly looking to intervene on the future in the present. Instead, alternate forms of knowledge, like the aesthetic forces in this article, are too crucial to the ability to secure. As I will show in more depth in a later section, varied literature has scrutinised the foundational role spatial-temporal staging,^15^ different emotions,^16^ visualisation^17^ and materiality^18^ play in the formation and performance of exercises and thus how crucial these aesthetic forces are to the security apparatus.

Alongside showing how exercises assess and develop protocol, the article contributes to this literature by arguing that exercises imagine future emergencies through acting as a site for the enmeshing of different aesthetic forces. To address both the aesthetic forces which collectively constitute the exercise and how exercises assess and develop protocol, I engage with Karen Barad’s^19^ notion of post-human performativity. An argument echoed now across cultural geography,^20^ Barad states that performativity does not need to be confined to an analysis of speech acts but can instead account for the active enrolment of human and non-human agents together in knowledge creation processes. Barad stresses how the world is iteratively made and remade by the interactions and intra-actions between an array of heterogeneous human and non-human forces. To imagine emergencies, the exercise functions through relations which are made and remade between different aesthetic forces as the exercise itself is acted out and performed. Drawing on Barad’s work, I argue that the relations between multiple agential forces, from the digital technologies which give the event its audio-visual character, the material space of the exercise site to the corporeally intense acting out of the exercise, are integral to the invocation of future emergencies in exercises.

Barad’s departure from understanding performativity merely as a concept that addresses action within the constraints of language allows also for an appraisal of how exercises work to assess and develop protocol too. Synthesised with Alexander Galloway and Eugene Thacker’s work on protocol,^21^ Barad’s work allows for an understanding of protocol not simply as written instructions to which responders in an emergency comply. Instead, protocols are the product of, and actively have a role in governing, the particular intersections between aesthetic forces which invoke the future emergency in the exercise. This is because exercises work specifically to enact protocol and, as such, the aesthetic forces evident in exercises focus on making protocol an object of strategic appraisal. The intersections between aesthetic forces in exercises are thus centred on testing and producing protocol. As a product of exercises, protocol is also emphasised in this article as contingent, as something made and remade through its continual assessment and development in exercises over time.

This article is structured as follows. Drawing on Galloway and Thacker, I first characterise protocol as a phenomena that enables the enactment of decentralised, networked power relations based on their capacity for modulation, adaptation and contingency. I also show how protocol can be understood as both a product of human-non-human relations and as a mode of action which consolidates these relations. I then situate FRS exercises within a detailed account of the different aesthetic forces discussed in previous literature on exercises. In turn, I argue that to properly conceptualise the design and performance of exercises, along with how they enact protocol, emphasis needs to be placed on an account of post-human performativity as advanced by Barad. After that, I break down the forms of protocol prevalent in the FRS and describe the design and performance of exercises by the aesthetic forces they configure and enrol to imagine future emergencies. In the conclusion, I show how both pre-existing literature on protocol and Barad’s account of post-human performativity are crucial to explaining how exercises work to assess and develop protocol but also open up to interrogation wider questions about the character of risk-based security organisations.

## Exercises: performing emergencies in the contemporary security apparatus

In *Protocol, Control and Networks*,^22^ Alexander Galloway and Eugene Thacker examine the role of protocol in relation to the operation of biological and Internet systems. These two systems are described as decentralised, horizontal networks comprised and underpinned by the relations between nodes. Protocol is fundamental to the operation of such network systems. The authors describe protocol as ‘the conventional rules and standards that govern relationships in networks’.^23^ An example of protocol in relation to the Internet would be the algorithmic rules which allow communication between computers, whereas biological systems protocol would be the information which circulates between cells in an organism or across an environment. Operating in networks specifically, the authors understand protocol as emblematic of, and integral to, the orchestration of a particular configuration of power relations premised on decentralisation. Networks have no centre. Rather than a vertically structured hierarchy, networks are constituted through nodes disparately scattered across a space. Nodes are ‘interconnected but autonomous entities’^24^ which, through protocol, are enrolled into the wider functioning of a system.

Along with being decentralised, networks too are characterised by their fluctuating nature and are driven by the necessity to change and be reconfigured over time. In relation to the Internet network, for instance, lines of communication between two people will be established one minute and then closed the next when the conversation is over. Alternately, a reduction in bandwidth capacity for the transferral of information will mean that an alternate conduit will be found, taking the information through a different place. As the conditions governing the relations between nodes upon which networks rely, protocol too must be flexible and adaptive. In naming the virtues of protocol, Galloway and Thacker argue that protocol operates in a way that is contingent and that ‘while protocol is universal, it is always achieved through negotiation (meaning that in the future, protocol will be different)’^25^ Owing to the contingency cast across the network, protocol must be made and remade over time. It must, in other words, also be contingent.

Galloway and Thacker not only posit protocol within a wider configuration of power and describe its iterative and ever emergent dynamic but also explore what protocol actually consists of. Facilitating and governing relations, protocol captures the material substance of nodes in a network (whether that is conversation content or, in the case of biological networks, DNA) and renders this material compatible with wider immaterial structures that enable relations to be forged. Conversations are turned into meta-data and feed into wider databases while DNA is posited within double helix structures, for instance. Combining material objects with their articulation as abstract immaterial code, the authors observe that protocol consists of information which they describe as something ‘immaterial and materialising, abstract and concrete, an act and a thing’.^26^ In their description of its contents, Galloway and Thacker demonstrate that not only does protocol facilitate and govern relations between different nodes; rather protocol is the product of the enmeshing of an array of heterogeneous forces. Furthermore, that protocol comes into sight when it acts to consolidate relations between such forces.

The protocol of networks the authors discuss bears much semblance with protocol enacted in the FRS. Specifically, with regards to the aims of this article, protocol is understood as inscribing fire governance with a flexible, adaptive capacity; enabling a set of prescribed actions to be made and remade in a way that is contingent upon new renditions of the fire emergency of the future. Furthermore still, Galloway and Thacker paint a picture of protocol as performative. Protocol is manifest in the effects of its actions; namely to consolidate and sustain relations. But the performativity of protocol is underpinned not just by how it facilitates relations but how it is produced through the enmeshing of materially heterogeneous agents. Both the contingent and performative nature of protocol will be returned to with regards to Barad and post-human performativity later in this section.

In relation to the FRS, protocol is shaped, tested and advanced through exercises. As techniques used to secure and govern emergencies, exercises have been the topic of varied discussion across literature spanning not only cultural geography^27,28^ but international relations,^29^ anthropology^30,31^ and the performative arts.^32^ But how in this literature are exercises seen to make present future emergencies which allow protocol to become an object of appraisal? The answer to this question is through a range of different aesthetic forces; knowledges and technologies which render the future emergency accessible in different ways in the present. In their work on exercises in the context of UK Civil Contingencies, Adey and Anderson claim that exercises operate and bring to life future emergencies by invoking the emotions and senses which are, and will likely be, provoked in emergency responders in the real time unfolding of emergencies. Exercises replicate the affective atmosphere experienced in an emergency. These atmospheres are constituted by and invoked through calling forth an array of heterogeneous, conflicting and temporary emotions within the emergency response professionals involved; including laughter, fun and mockery but also panic and confusion. It is contended by the authors that these emotions also underpin the temporal structure of the exercise. The provocation of different senses creates interruption and disruption between the present performance of the exercise and the future that the exercise purports to represent, allowing a temporality to emerge which ‘is neither present nor future’.^33^ The authors refer to this time as an ‘interval’^34^ which breaks the progressive continuum of present -future. Through the stoppages this interval causes, exercise participants are able to stop and consider mitigation techniques and to prepare resources for a future yet to unfold.

Aradau and Van Munster’s^35^ take again stresses the temporal actualisation of exercises. But their emphasis is on how the discursive description and narration of the exercise works to evoke the temporal unfolding of the emergency imagined. Exercises invariably start, the authors claim, with a description of the time immediately before the emergency and then develop onwards through different stages. As the authors show, exercises also outline the physical surroundings in which the emergency is about to take place; whether that is a racecourse, an international conference at a hotel or a village summer fete to use the authors’ examples. The temporal structuring of the exercise is thus always conjoined with and embedded in spatial referents. It is through the discursive narration of the exercise that the space-time of the future emergency is invoked.

Exercises appear in the two works above as a maelstrom of senses, but a maelstrom nevertheless organised by spatial-temporal coordinates. The harnessing of emotions and senses through the imagined space-time of emergencies is accompanied also by discussion of the realism of exercises across literature. I raise the matter of realism not to submerge this article into a discussion of the accuracy of events portrayed in exercises but to indicate how the issue of realism reveals other sensual and aesthetic forces at play. For example, discourses of realism, to return to Adey and Anderson’s work, appear in the form of exercise participants’ commentary on the plausibility of the exercise. Plausibility acts as a condition of regulation to ensure the events portrayed are not too fantastical, do not stretch too much the acknowledged normative similitude emergencies of the same type share. As the authors note, ‘events . . . designed into scenarios (exercises) is very rarely about the imaginary of the “unimaginable”’.^36^ What is fantastical and what is plausible, importantly, is an issue arbitrated upon by the memory and experiential knowledge of those involved in various aspects of the exercise. Exercises thus ‘anchor the interval of emergency in “realistic” details *that resonate with players’ tacit and codified knowledges* of the area’.^37^ In encountering the issue of realism, another sensual force has been revealed in its enrolment within the exercise. The plausibility of the exercise depends on its ability to forge connections between the event it portrays and the experiential knowledge and memory of its participants. This manoeuvre shows, moreover, that exercises are not only created and performed through invoking a set of sensibilities in their participants but must also correspond to and derive from pre-inscribed sensual registers accrued over a life time in emergency response. Along with space-times and a variety of senses, the memory and recollection of exercise participants feeds into the rendition of future emergencies in the exercise.

In some cases,^38^ the capability of exercises to offer a realistic impression of future emergencies is intrinsically tied to the question of what material props are found in the exercise. Coming in the form of shrapnel,^39^ or the remnants of humans,^40^ for instance, material objects render the future emergency imaginary concrete. At least in pre-existing literature, these material props are also the principle means by which the emergency can be invoked in its audio and visual output. As Davis notes, whether ‘the wail of a siren, shoes getting in the way, rags for curtains and tablecloths; an upturned table with a spilled vase of flowers or deck of cards’,^41^ it is the materialities enrolled into the exercise which afford the exercise its capacity to replicate the sights and sounds of the scene of an emergency.

The coordination of space-times, senses, matter, the audio-visual and the insertion of other aesthetic forms of knowledge like memory. This ensemble of forces, which can be traced across exercises in a number of contexts, are also evident in the design and performance of exercises in the FRS as I will go on to demonstrate. Authors in those works engaged with above also note how different forces coagulate and overlap with one another. Adey and Anderson thus describe how emotional states punctuate the exercise temporally while Aradau and Van Munster argue that the spatial and temporal qualities of the exercise are inseparably bound to one another. But attention has yet to be paid to how all the forces circulating in the exercise are enmeshed with one another and how this set of complex relations are made and remade in order to structure, condition and bring to life the emergency that the exercise represents and simulates. Neither have exercises been tied to the assessment and development of protocol which will prepare and structure response for future emergencies.

This article addresses these gaps by examining both how future emergencies are imagined through the relations between aesthetic forces in exercises and the enactment of protocol through understanding exercises as a site of what Karen Barad calls post-human performativity. As it appears as a concept across geography and social sciences more generally, Barad claims that performativity has been reduced in its potential efficacy to the framing and analysis of speech acts. In conventional understandings of performativity, speech acts bring their referent object into being through the representations they offer. Not only in other words does language operate to describe reality but rather in its utterance it actively participates in the course of making reality. According to Barad, performativity could be extended and applied to explore how things come into being through the act of making and remaking relations between human and non-human agents. Speaking specifically in relation to the production of matter, studying how these relations are established and consolidated works to give ‘matter its dues as an active participant in the world’s becoming’.^42^ The relations found might, as Barad argues, come in the form of intra-actions where configurations form and occur within a circumscribed space. Or interactions might be found where wholly separate spheres of activity come into contact with one another. Overall, however, the point is that performativity here refers to the act of making and remaking relations between a heterogeneous array of agencies and the production of matter on the basis of these relations.

To be applicable to how emergencies are imagined in exercises, post-human performativity must extend beyond matter to how matter relates to a number of other above-mentioned forces such as audio-visual technologies, the sensibilities of human participants and relations between human participants. What the notion of post-human performativity offers is a means to conceptualise how these forces relate to and enmesh with one another. As Barad would note, they enmesh with one another in the process of the exercise’s undertaking. Through the exercise’s performance, in other words, various aesthetic forces encounter one another and collectively imagine the emergency. Performance equates to the enrolment of one aesthetic force into another and the consolidation of relations between things through the doing of the exercise. In the performance of the exercise, the different aesthetic forces mentioned above – emotions, space-times, materialities and digital technologies – come together to offer renditions of future emergencies which can be prepared for in the here and now.

Barad’s take on performativity has found traction both in cultural and political geography^43^ and in critical security studies.^44^ Aradau, for example, has read the securitisation of critical infrastructure through Barad’s emphasis on the efficacy of non-human forces which shape and condition governmental interventions. For Aradau,

Critical infrastructure is not just the result of a complex assemblage of social practices and values . . . but it emerges as an object whose materiality has both enabling and constraining effects and what can be said and done to secure it.^45^

For Aradau, Barad’s approach makes way for understanding how the deployment of critical infrastructure protection is contingent in part due to the autonomy of the material things to which it is oriented.

Read through Barad’s understanding of performativity, critical infrastructure protection shares with protocol a contingency dynamic, as evidenced in Galloway and Thacker’s reading of protocol. As was noted above, protocol is contingent owing to the contingency of the networks to which it is attendant and operable within. The necessary making and remaking that protocol goes through is not the only line of similarity that can be drawn between protocol and Barad’s post-human performativity however. Rather, protocol is rendered manifest and produced through relations between heterogeneous material forces. The consolidation and enactment of protocol relies on its emergence on the grounds of the enmeshing of different agencies. Similarly, in the FRS as I will show now, protocol is produced and re-produced through exercises which imagine future emergencies through coordinating different aesthetic forces.

## Protocol in the FRS

The set of protocols deployed by the FRS to plan response to fire emergencies of the future considered in this article are collectively known as ‘incident command’.^46^ Within incident command, generally, are more specific risk assessment protocols. There are three types of risk assessment protocols. First, a ‘generic risk assessment’^47^ exists which sets out and guides response in the event of a fire in particular kinds of space. Generic risk assessments address a vast range of different spaces in which fires might unfold including high rise buildings,^48^ industrial plants^49^ to farms^50^ and in tunnels.^51^ Risk assessments involve identifying the specific hazards, dangers and difficulties tackling fires in different spaces might present. With regard to the generic risk assessment for fires at a height, the specific risks evident here include the possibility of structural collapse and the rapid spread of fire due to the internal lay-out of the building. In turn, generic risk assessments offer guidelines for how to respond to fires taking place at such sites including what kinds of equipment to use, the distance from which cordons around the building should be established and ways by which to evacuate people.

Showing the need to adapt and create new protocol for fire emergencies that have been newly thought up, and the contingency of protocol in general, the other two forms of protocol work to provide parameters for tailoring response actions not just to the type of space in which the emergency occurs but to the specificity of the unique fire event itself. Dynamic risk assessments seek to supply guidelines for strategic decisions made in adaptation to the unique, complex and unforeseeable set of developments which might characterise an individual incident. What this dynamic risk assessment refers to, according to one member of staff I interviewed, is how strategy might be implemented by fire fighters ‘thinking on their feet’ where an incident’s unfolding transcends the limits of established protocol. The last form of protocol enacted is called ‘analytical risk assessment’.^52^ Actions taken under the structure of analytical risk assessment show a similarity to dynamic risk assessments as they offer guidance on how strategy might be redeployed out of a necessity to adapt to the peculiarity of an incident which is unaccounted for in existing guidance. In contrast to dynamic risk assessment, however, analytic risk assessments concentrate also on how to formulate coordinative response between fire fighters. Analytical risk assessments might involve, for instance, outlining alternate ways in which communications between fire fighters at different areas within the site might be engendered when conventional forms of communication have broken down.^53^

As I will turn to demonstrate in the next section, ‘The gift of sound and vision: designing the exercise’, exercises work to assess pre-existing protocol and develop new forms of protocol. Both the assessment and development of protocol is achieved in exercises by pushing the limit of pre-scribed protocol. To do so, exercises stage and imagine types of fire emergencies yet to be addressed by pre-existing protocol. In relation to the assessment of protocol, exercises present new emergency scenarios in order to gauge the applicability of protocol beyond the type of fire incident it was formulated around. If *assessment* of protocol is about gauging the applicability of protocol, the *development* of protocol is actualised by recording the action of participants which exceeds pre-established protocol. Observations of participants’ actions in exercises are then fed back to generate wholly new forms of response protocol altogether.

## The gift of sound and vision: designing the exercise

In this section and the next, ‘Doing the exercise, performing the future’, I present empirical material generated from 6-months research in the FRS. The research involved non-participative ethnographic observation of several exercises taking place in an FRS headquarters in the north east of England. The research also involved semi-structured interviews with members of the FRS’ Learning and Development team who design exercises and coordinate their performance. Drawing on material generated from this research, this section considers two fundamental elements of exercises. First, I outline where the ideas for the emergency scenarios depicted and performed in exercises emerge from. I then turn to describe how these ideas are rendered audio-visually. Both thinking up and rendering emergencies audio-visually are elements in the process by which exercises are designed.

Where do the ideas for emergencies found in exercises actually come from? When asking this question, literature which takes exercises in their incarnation as a technique for securing large scale events suggests that exercises have become especially important post 9/11.^54^ This is because after 9/11, there was a concerted drive by governments across the world to imagine potential emergencies which had heretofore been unthinkable.^55,56^ After 9/11, exercises thus became a pivotal means to speculate and act out futures which could not be captured by more conventional risk analysis techniques such as data mining or risk mapping. In the FRS, exercises indeed are efficacious because of their ability to harness and mobilise forms of knowledge considered unconventional in governing risks. But the answer to the question of where the ideas for emergencies found in exercises bears a more banal answer in the case of the FRS than in cases where larger contingencies are imagined. As explained to me by the Training Co-ordinator of the FRS, ideas for emergencies in exercises simply emerge from experienced fire fighters living within the area that they work in. Fire fighters will notice a specific building or site in their local area which bears within it the potential for an emergency fire scenario which the FRS may be unprepared for. In turn, the process of designing an exercise around the emergency sensed will be instigated.

This insight immediately suggests the crucial role of experiential knowledge in designing exercises. The ideas for exercises around specific emergencies spring forth not from sophisticated digital analytics, for instance, but from the ability of fire fighters to draw on their experience to speculate on the risks that could potentially unravel in certain built environments. The speculations of fire fighters are not grounded in a specific memory though. Instead, they are based on noting how potential futures exceed what is already planned for. The noting of gaps in preparedness is discovered by comparing fire fighters’ speculations about specific sites to existing knowledge of what protocols the FRS have in place. The potential dangers that a fire at a specific site might bear are checked and compared with the above-mentioned generic risk assessments and what are called Site Specific Risk Assessments (SSRIs). Referring to particular buildings in a locale, SSRIs comprise data on the material, infrastructural contents of a building and how many lives would be at risk should a fire occur. Showing how exercises function to test the applicability of protocol, fire fighter speculations on potential emergencies will be enacted as exercises if the emergencies imagined are adjudged to be unaccounted for in pre-existing guidelines such as generic risk assessments.

**Figure 1.**
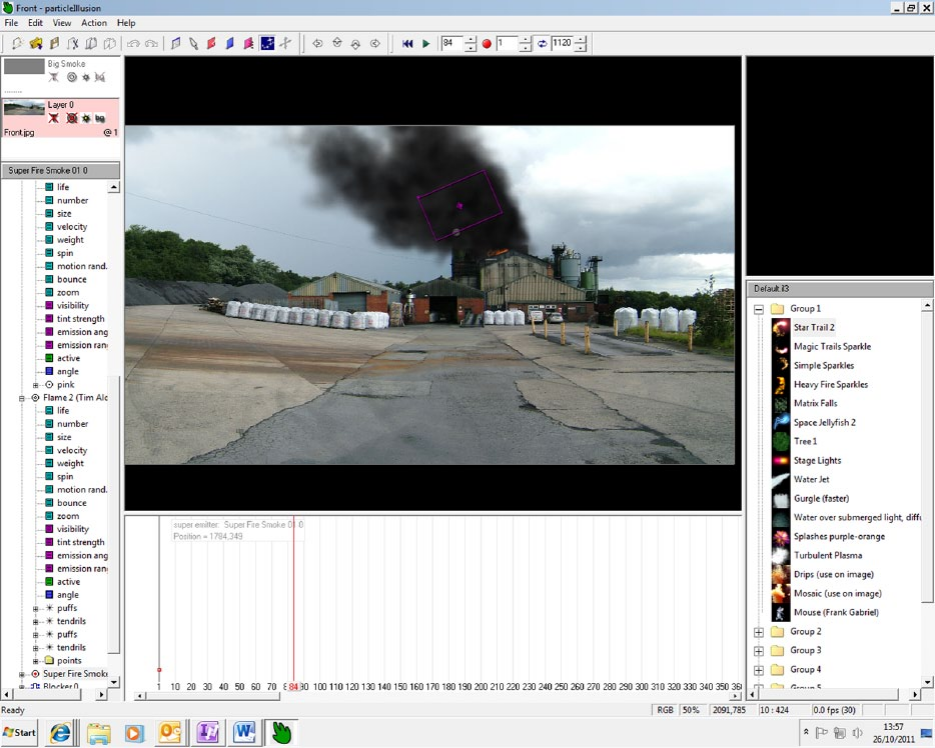
Particle Illusion’s audio-visual rendition of a fire emergency at a site as yet to be accounted for in FRS SSRIs.

Photos amassing to a 360 degree perspective of the site identified are taken. The photos taken act as a means to translate the experiential knowledge of fire fighters into actionable digitised data as photos are loaded up into software called Particle Illusion. Developed initially for cinema, Particle Illusion is a graphic motion technology which is used to offer an audio-visual representation of fire events. Through Particle Illusion, the photos taken at sites will be sequentially synthesised to form a panorama of the site at which the incident is imagined to have occurred. On top of this panorama, an in-built ‘fire graphics’ component in Particle Illusion presents flames and smoke, visualising the extent of fire and the gradual deterioration of the site (see Figure 1). Other data interject to visualise the fire. The force of fire will be envisaged by factoring in how wind speeds determine the velocity and spread of fire. Furthermore, the so-called ‘weight’ of the fire is represented according to the interaction the fire would have with the material contents found at the site. The sound of fire will be inserted through an audio component. Overall, an audio-visual clip of around 30 seconds will be created, looped and adjoined to clips showing the initial intensification of fire and its decrease to intimate eventual control.

Through Particle Illusion, future fire emergencies are rendered both audibly and visually. It is important to note that this cinematic rendition of future emergencies represents a filtering of the speculations of fire fighters into digital technologies. Futures imagined are the result thus of a particular enmeshing of experiential knowledge and memory with audio-visual technologies. However, a much wider set of knowledges and technologies are interwoven and coordinated in imagining emergencies. To outline how this conglomeration of aesthetic forces is enacted, I now turn attention to how the exercise is actually performed.

## Doing the exercise, performing the future

**Figure 2.**
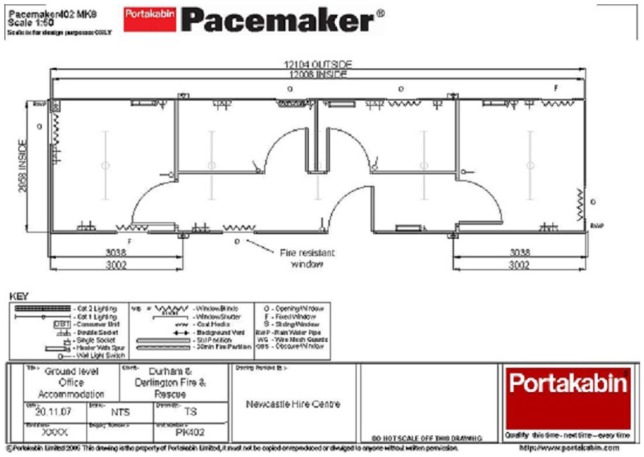
A blueprint showing the spatial arrangement of the syndicate room in which exercises take place.

The next place one would see the audio-visual renditions Particle Illusion makes of future fires would be in the control room of the FRS’ Incident Command Centre (ICC). The ICC is where exercises are held. The ICC exists across two storeys. The bottom floor features the syndicate room (see Figure 2). Divided into four spaces by partitions, the syndicate room is where participants act out the exercise itself. On the top floor are two rooms. One of these rooms is called the briefing room. Before the exercise starts, participants are gathered in the briefing room and given an initial description of the fire imagined to be taking place. Adjacent to the briefing room is the aforementioned control room. The control room has multiple purposes. This room acts as a kind of backstage from which exercises are orchestrated and coordinated. It is from here that the audio-visual representations of emergencies generated in Particle Illusion will be controlled and projected into the syndicate room. Furthermore, the control room is used to scrutinise the strategisation, decision making and activity of those taking part in the exercise. The control room consists of 12 screens covering two walls. Four screens are laid out across the bottom of the wall. Running upwards, another two layers of four screens are found. The bottom row of screens are the ones in which the audio-visual elements of the emergency imagined are uploaded and projected into the syndicate room. The additional two layers of screens are connected to closed circuit television (CCTV) cameras in syndicate rooms. The first layer of screens focusses on the activity in each syndicate room while the second row monitors the movement between each syndicate room, giving a seamless perspective on the movement across the whole of the bottom floor of the ICC. Further to CCTV connections, the control room and the syndicate room are connected by microphones. Not only can the action and movement of exercise participants be monitored visually but the negotiations between participants in making decisions and enacting strategy can be audibly recorded.

Set in the briefing room, exercises start with participants being confronted by a text-based description of the incident imagined to have occurred. Variables of the fire incident are outlined including the location of the site, the potential material contents of the site and the possible risk to human life. Operative roles will be designated to exercise participants in this moment. The Incident Commander, who will have overall authority in strategic decision making about how to attend to the fire, along with fire fighters, fire engine drivers, Watch Managers and Crew Managers are all roles bestowed on participants. The delegation of these roles and duties simultaneously acts to further structure the timeline of the scenario in a way assimilated with real events. The Incident Commander will be the first to arrive at the scene with a driver and two fire fighters. After an indefinite amount of time, the Watch Manager will arrive with more fire fighters and lastly, the Crew Manager with additional resources.

Once these roles are distributed, exercises simulate the transit time to an event, imagining the route does not involve simulation of driving to an event but using the spatial divisions of the ICC itself. Before arriving at the syndicate rooms, participants are stopped and supplied with information on the fire that could be expected when travelling to an incident site. The first element of protocol assessment takes place here. In driving to an event, fire fighters should be gathering information on the fire and, according to how the risks present can be made sense of in relation to generic risk assessments, decisions should be made such as what kinds of equipment will be needed.

The arrival at the scene of an incident equates to arriving in the first syndicate room. Entering the syndicate room, participants encounter the fire graphics produced by Particle Illusion. Each of the four syndicate rooms presents a different angle on the site at which the fire is taking place. As the rooms are partitioned from one another, views of the fire are limited. The partitions in the syndicate room thus become enrolled in the exercise and take on symbolic power as structures which block the fire from view. Arriving at the scene first, the Incident Commander confusedly navigates through these rooms, gradually attaining a 360 degree perspective on the site. In moving around this imagined emergency site, participants will seek to extrapolate the cause of the fire, its preliminary location in the site, what potential hazards can be identified and if any human beings are present. In turn, decisions either based on pre-existing protocol or exceeding such protocol are made which direct the FRS’ response strategically.

From here, the emergency exercised develops through the deployment of what are known as injects. Engendered by the control room, these injects amount to different stages which are imagined to occur within the emergency being acted out. Over the course of an exercise, injects include the audio-visual unfolding of the emergency. Here, images are projected into the syndicate rooms. The fire may be visualised to spread to different locations within the site, coming into contact with new types of materials. At the same time, different sounds will be introduced to intimate the development of the fire. Showing further the enrolment of the material site of the exercise into the emergency imagined, explosions and the collapse of structures, for instance, will be injected and bounce on and across the walls and partitions of the syndicate room. These injects might also take human form with the introduction of new role players in the exercise. A host of different members of the public could be introduced during the scenario. According to the Training Co-ordinator, this has in the past ranged from drunken football fans to angry farmers demanding answers while their livelihood is being swept away in the flames.

The timing of the deployment of injects and what they convey in terms of the development of the emergency are organised by a reciprocal tie established between the control room and the syndicate room. As a control room operator remarked, ‘you sort of . . . use your timescale dependent on the individual (exercise participant) to get more out of the exercises’. On audio-visual injects, the operator stated that injects involve ‘ramping up or ramping fire down depending on their (exercise participants) actions’. As much as staff taking part in scenarios will make decisions in a way that is conditioned by injects from the control room, the injects themselves will intervene in a way that is conditioned by the decisions made by staff taking part in the exercise. As injects direct the development of the emergency, the temporality of the exercise is co-produced by the actions of distinct, albeit inter-related, actors in the exercise site and their observers above.

The emergency that the exercise portrays is structured around a dynamic timeline which is continually made and remade by the negotiations between the two on-going hubs of activity and decision found in the ICC: the control room and the syndicate room. Across the duration of the exercise, an incident’s development was described by the ICC Technician as ‘very flexible’ and ‘very fluid’. In alignment with Adey and Anderson’s observations, the shifting temporality of the exercise replicates and invokes the contingent and inchoate temporality of emergencies in their real-time unfolding. The following example was provided to explain how the contingency of emergencies is inscribed into the temporal structuring of exercises. Usually for response times, firefighters can expect ‘a second appliance (to) come in two minutes’ after the first appliance has arrived. The Training Co-ordinator goes on to note how ‘we might hold those appliances back’ in exercises to increase the complexity of the incident and to examine how staff responding will negotiate with such a development. It is thus not only a set of harmonious reciprocal responses that coordinate the making and remaking of the temporal structuring of the exercise. Instead, slippages in this relation are purposively enacted to create a sense of the contingency which is supposed to organise the life of real emergencies. The performative enrolment of one aesthetic force into another is not only enacted through close attachments between one thing and another, with the material space interweaving with the audio-visual rendition of fire, for instance. Rather, gaps and junctures in these lines of relation are exploited to invoke the contingency of the emergency. Creating this uncertainty around how the emergency will develop, exercise participants can be assessed in terms of how they use dynamic and analytical risk assessments. In other words, how they operate in excess of prescribed protocol. These actions, which exceed protocol, can in turn be recorded and formulated into new protocols.

In seeing the performance of the exercise as the matter of enrolment between different aesthetic forces, the ‘sense’ of contingency invoked does not just appear in the flat, if rupturous, temporal structuring of the exercise. Instead, it is emergent at the affective and emotional register of the exercise. As Adey and Anderson were seen to claim, the instigation and sharing of a particular moment of laughter, of hope or panic between exercise participants help to evoke the emergency in its simulated form. In particular, exercises in the FRS proceed through provoking a confusion in their participants which derives from the event portrayed breaking with what might be expected in a similar events’ representation in generic risk assessments. Many of the aspects of the exercise described already attest to this fact. For instance, partitions are enrolled into the imagining of the emergency specifically to block the Incident Commander’s vision and knowledge of the fire taking place. Similarly, injects indicating the arrival of additional personnel or resources will be delayed. The intermixture of different forces in the exercise work to provoke emotional reactions from participants which in turn circulate and become part of the exercise’s simulation of an emergency.

## Conclusion: exercises, post-human performativity and protocol

Exercises are an ensemble of heterogeneous human and non-human agencies, what I have called aesthetic forces. Following Barad, this complex set of forces enact relations with one another and, in doing so, coordinate to make present future emergencies. The digitally rendered sights and sounds of an emergency, along with its textual, discursive narration, are invested into and interweave with the physical space of the exercise site. In doing so, the material confines and features of the site are too enrolled and enwrapped into the staging of the emergency. The space between rooms in the ICC is thus crucial to invoking the transit time to an emergency for instance, while partitions which divide the syndicate room stand in for different angles from which the fire might be viewed and experienced. Having inscribed upon them different audio-visual and textual effects of the emergency, the physical and objective space of the exercise site takes on properties beyond its mere materiality. They are invested instead with a symbolic power as props amidst which the emergency unfolds.

But the exercise described above affirms also that human action is central to the performance and harnessing of these relations along with their mutual co-rendering of future emergencies. Human mobility might reinforce aesthetic forces already congealed with one another. For spaces in-between rooms to evoke the transit time to an emergency requires the stoppage of human movement from one room to the next. Similarly, only human sight can bring to the fore difficulties in attaining a full view of the fire due to the use of partitions in the exercise site. Human agency not only reinforces relations between aesthetic forces which are performed and established before the immersion of human actors. Although partitions work to fragment the view of the fire, human actors introduce here sensual outputs such as confusion which circulate across the exercise site. Furthermore, the exercise is underpinned by a temporal structure which emanates from the perpetual interactions between exercise participants and those coordinating the exercise. The enrolment of senses and temporal structuring into the wider set of non-human aesthetic forces at play mobilise within the exercise a sense of contingency which is deemed integral to common understandings of emergencies. Ultimately, human performance actively adds new layers of complexity to the emergency being acted out by introducing new forces into the unfolding of the exercise.

Exercises also work to assess and develop new forms of protocol. As Galloway and Thacker describe, protocol is contingent. It is subject to flux and renegotiation. Exercises might be said to supply protocol with its contingent nature by both testing protocol and pushing the FRS to establish new protocol which adapts to new forms of fire emergency incubating in the minds of emergency responders. Affording protocol with its contingency is witnessed first at the design stage, where future emergencies are only mobilised as exercises if they bear the potential to stress-test, exceed and surpass protocols which have already been established. That is, if the emergencies imagined show a gap in the preparedness of the FRS. But protocol is also assessed and developed through the performance of the exercise itself. Confronted by an atmosphere characterised by its sense of contingency, emotions which circulate and conjoin with the audio, visual and material output of an emergency, the performance of firefighters in dealing with this situation is recorded and forms the foundation for the development of new protocol.

As protocol is so intimately tied with the exercise both in terms of assessing its applicability and its capacity to be developed anew, Galloway and Thacker can also be followed in claiming that protocol is a product of, and has a role in mediating, the relations set up between materially heterogeneous forces through which exercises imagine future emergencies. To reiterate, the authors showed how, in relation to networked systems, protocol facilitates and governs relations between disparately scattered nodes. Protocol has this consequence too in exercises because the aesthetic forces assembled imagine emergencies which are oriented towards the assessment and development of protocol. As the object which is sought for strategic appraisal in exercises, protocol is central to the mutual harnessing of different forces which make future emergencies present in exercises. However, Galloway and Thacker also stress that protocol consists of information, information which combines material substrates with immaterial language and rules. Protocol is thus built-up of and produced by the enmeshing of the thoroughly heterogeneous maelstrom of forces which it brings together in the first place. The same dynamic whereby protocol not only plays a role in establishing intersections between human and non-human agents but is a result of their interplay can be observed in exercises in the FRS. Assembled initially to make protocol an object of strategic appraisal, the aesthetic forces which underpin exercises work to present emergencies which cannot be attended to through pre-scribed protocol. These forces not only gather around protocol, rather they present situations in which fire fighters will necessarily improvise and act in excess of protocol. In turn, new protocol will be established. Protocol is thus the product of the interweaving of different aesthetic forces which make present future emergencies in exercises whilst being the node around which aesthetic forces come together.

With the turn towards anticipatory, risk-based modes of governance not just in relation to the governance of fire but also in emergency response in general the array of techniques, knowledges and technologies used by such organisations has been extended. No longer, for example, is fire known merely through probability statistics or attended to through the mobilisation of resources in the moment of their chance occurrence. Exercises and protocol are prime examples of the new phenomena which have risen to prominence in an age of securing the future. Understood as performative devices in the post-human sense that Karen Barad outlines, this article makes sense of, and provides critical tools for understanding, the life of exercises and how they enact protocol. Both protocol and exercises, as has been argued, are relationally based; their bringing into existence and deployment relies on the play of intersections between diverse forces. As the article has stressed, it is important to understand how, and with what immediate consequences, relations between these forces are configured. This might mean exploring, for instance, how audio-visual renditions of the emergency generated through computer software interact with the physical space of the exercise site. But protocol allows for an insight too into the kinds of intervention which emerge where security apparatuses orient themselves towards the future. Protocol, as Galloway and Thacker would have it, are premised on contingency; its capacity to be renegotiated according to the futures acted out in sites such as the exercise are one of protocol’s main attributes. As one of its central components, protocol helps to produce an FRS that is itself iterative and flexible; acclimatising to contingencies of the future it seeks to know and perform in exercises.

## Notes

1. UK Act of Parliament, *Fire and Rescue Services Act*, 2004.

2. UK Act of Parliament, *Civil Contingencies Act*, 2004.

3. B.Anderson, ‘Preemption, Precaution, Preparedness: Anticipatory Action and Future Geographies’, *Progress in Human Geography*, 34, 2010, pp. 777–98.

4. M.de Goede, *Speculative Security: The Politics of Pursuing Terrorist Monies* (London: University of Minnesota Press, 2012).

5. P.O’Malley, *Risk, Uncertainty and Government* (London: Glasshouse, 2004).

6. Communities and Local Government, *Fire Service Manual: Incident Command*, vol. 2, 3rd ed. (London: Communities and Local Government, 2008).

7. For a detailed account of how preparedness sits in relation to other anticipatory strategy see Anderson, ‘Preemption, Precaution, Preparedness’.

8. L.Amoore, *The Politics of Possibility: Risk and Security Beyond Probability* (Durham, NC: Duke University Press, 2013).

9. M.de Goede and S.Randalls, ‘Precaution, Preemption: Arts and Technologies of the Actionable Future’, *Environment and Planning D: Society and Space*, 27(5), 2009, pp. 859–78.

10. L.Berlant, *Cruel Optimism* (Durham, NC: Duke University Press, 2011).

11. C.Aradau and R.Van Munster, *The Politics of Catastrophe: Genealogies of the Unknown* (London: Routledge, 2011).

12. L.Manovich, *The Language of New Media* (Cambridge: MIT Press, 2001).

13. C.Aradau and R.Van Munster, ‘The Time/Space of Preparedness: Anticipating the ‘Next Terrorist Attack’’, *Space and Culture*, 15(2), 2012, pp. 98–109.

14. S.J.Collier, ‘Enacting Catastrophe: Preparedness, Insurance, Budgetary Rationalization’, *Economy and Society*, 37(2), 2008, pp. 224–50.

15. Aradau and Van Munster, ‘The Time/Space of Preparedness’.

16. P.Adey and B.Anderson, ‘Affect and Security: Exercising Emergency in UK Civil Contingencies’, *Environment and Planning D: Society and Space*, 29, 2011, pp. 1092–109.

17. Collier, ‘Enacting Catastrophe’.

18. T.Davis, ‘Stages of Emergency: The Casualties Union’, *Modern Drama*, 46(2), 2003, pp. 151–81.

19. K.Barad, ‘Post-Human Performativity: Toward an Understanding of How Matter Comes to Matter’, *Signs: Journal of Women in Culture and Society*, 28(3), 2003, pp. 801–31.

20. See S.Whatmore, ‘Materialist Returns: Practising Human Geography in and for a More than Human World’, *Cultural Geographies*, 13(4), 2006, pp. 600–9.

21. A.Galloway and E.Thacker, ‘Protocol, Control and Networks’, *Grey Room*, 17, 2004, pp. 6–29.

22. Galloway and Thacker, ‘Protocol, Control and Networks’.

23. Galloway and Thacker, ‘Protocol, Control and Networks’

24. Galloway and Thacker, ‘Protocol, Control and Networks’

25. Galloway and Thacker, ‘Protocol, Control and Networks’

26. Galloway and Thacker, ‘Protocol, Control and Networks’

27. Adey and Anderson, ‘Affect and Security’.

28. P.Adey and B.Anderson, ‘Governing Events and Life: Emergency in UK Civil Contingencies’, *Political Geography*, 31, 2012, pp. 24–33.

29. Aradau and Van Munster, ‘The Time/Space of Preparedness’.

30. Collier, ‘Enacting Catastrophe’.

31. M.Schoch-Spana, ‘Bioterrorism: Us Public Health and a Secular Apocalypse’, *Anthropology Today*, 1(20), 2004, pp. 1–6.

32. Davis, ‘Stages of Emergency’.

33. Adey and Anderson, ‘Affect and Security’.

34. Adey and Anderson, ‘Affect and Security’.

35. Aradau and Van Munster, ‘The Time/Space of Preparedness’.

36. Adey and Anderson, ‘Affect and Security’.

37. Adey and Anderson, ‘Affect and Security’.

38. Aradau and Van Munster, ‘The Time/Space of Preparedness’.

39. Adey and Anderson, ‘Affect and Security’.

40. Davis, ‘Stages of Emergency’.

41. Davis, ‘Stages of Emergency’.

42. Barad, ‘Post-Human Performativity’.

43. See Whatmore, ‘Materialist Returns’.

44. C.Aradau, ‘Security That Matters: Critical Infrastructure and Objects of Protection’, *Security Dialogue*, 41(5), 2010, pp. 491–514.

45. Aradau, ‘Security That Matters’, p. 429.

46. Communities and Local Government, *Fire Service Manual*.

47. Communities and Local Government, *Fire Service Manual*.

48. Communities and Local Government, *Generic Risk Assessments, GRA 3.1: Fighting Fires in Buildings* (London: Communities and Local Government, 2011).

49. Communities and Local Government, *Generic Risk Assessments, GRA 5.1: Incidents Involving Electricity* (London: Communities and Local Government, 2011).

50. Communities and Local Government, *Generic Risk Assessments, GRA 3.5: Fighting Fires in Farms* (London: Communities and Local Government, 2011).

51. Communities and Local Government, *Generic Risk Assessments, GRA 2.7: Rescues from Tunnels* (London: Communities and Local Government, 2013).

52. Communities and Local Government, *Generic Risk Assessments, GRA 2.7*, p. 68.

53. Communities and Local Government, *Fire Service Manual*.

54. Aradau and Van Munster, *The Politics of Catastrophe*.

55. M.de Goede, ‘Beyond Risk: Pre-Mediation and the Post 9/11 Imagination’, *Security Dialogue*, 39(2–3), 2008, pp. 155–76.

56. R.Grusin, *Premediation: Affect and Mediality after 9/11* (London: Palgrave Macmillan, 2010).

